# Unreliable Evidence: 2 Sources of Uncertainty During Perceptual Choice

**DOI:** 10.1093/cercor/bht287

**Published:** 2013-10-11

**Authors:** Elizabeth Michael, Vincent de Gardelle, Alejo Nevado-Holgado, Christopher Summerfield

**Affiliations:** 1 Department of Experimental Psychology, University of Oxford, Oxford OX1 3UD, UK; 2 Laboratoire Psychologie de la Perception, CNRS UMR 8158, 75006 Paris, France

**Keywords:** categorization, dorsomedial prefrontal cortex, fMRI, gain modulation, perceptual averaging

## Abstract

Perceptual decisions often involve integrating evidence from multiple concurrently available sources. Uncertainty arises when the integrated (mean) evidence fails to support one alternative over another. However, evidence heterogeneity (variability) also provokes uncertainty. Here, we asked whether these 2 sources of uncertainty have independent behavioral and neural effects during choice. Human observers undergoing functional neuroimaging judged the average color or shape of a multielement array. The mean and variance of the feature values exerted independent influences on behavior and brain activity. Surprisingly, BOLD signals in the dorsomedial prefrontal cortex (dmPFC) showed polar opposite responses to the 2 sources of uncertainty, with the strongest response to ambiguous tallies of evidence (high mean uncertainty) and to homogenous arrays (low variance uncertainty). These findings present a challenge for models that emphasize the role of the dmPFC in detecting conflict, errors, or surprise. We suggest an alternative explanation, whereby evidence is processed with increased gain near the category boundary.

## Introduction

Perceptual categorization involves quantifying sensory evidence and comparing it to a criterion or boundary (Green and Swets 1966; Ashby and Maddox 2005; Freedman and Miller 2008). For example, a particular color might separate “ripe” from “unripe” fruit for a foraging animal. Items with feature values close to this boundary elicit prolonged decision latencies, a delay that computational models attribute to the need resolve uncertainty—or conflict—between closely matched rival responses (Botvinick et al. 2001; Bogacz et al. 2006). In the human brain, this process has been attributed to a network including the medial prefrontal cortex, anterior insular cortex, and lateral parietal cortex (Botvinick et al. 1999; Huettel et al. 2005; Grinband et al. 2006).

While laboratory-based tasks typically involve categorization of an isolated visual stimulus, in the real world, decisions often require observers to integrate information from multiple sources (e.g., by averaging). For example, a hungry animal might decide where to forage by averaging the size or color of all the fruit in a tree. Critically, judgments about the average information in a multielement array are made in the face of 2 potentially orthogonal sources of decision-level uncertainty—those owing to the “mean” and “variability” of the feature values respectively. Placing a bet on a soccer match, one might consider the average skill level of all the players in a team. However, picking a winner will be challenging when mean ability in the 2 teams is well matched, or when skill levels are variable (e.g., one team has excellent strikers but a weak goalkeeper). Both the mean and variance of evidence can influence error rates and prolong decisions (de Gardelle and Summerfield 2011), suggesting that human observers can compute such summary statistics about visual stimuli (for a review, see Alvarez 2011). However, little is known about the brain mechanisms that underlie these computations.

Here, thus, humans undergoing functional magnetic resonance imaging (fMRI) categorized an array of 8 elements according to either its average color or shape. Critically, we manipulated independently the parameters of the distributions from which feature information was drawn, thereby varying uncertainty due to the mean (*U*_M_) and variance (*U*_V_) of the evidence on both behavior and brain activity. When the array mean was closer to the category boundary (increased *U*_M_), BOLD activity in the dorsomedial prefrontal cortex (dmPFC) and anterior insular cortices (AINS) increased as previously described (Huettel et al. 2005; Grinband et al. 2006). Remarkably however, and contrary to our predictions, increasing the variability (thereby increasing *U*_V_) had the opposite effect, with dmPFC and AINS showing relatively “decreased” BOLD responses when evidence variability was increased, that is, when the feature values were more heterogeneous (and performance declined). These findings are hard to explain if the function of the dmPFC is to monitor for uncertainty (Botvinick et al. 2001), predict errors (Brown and Braver 2005), or scale with time-on-task (Grinband et al. 2011). We suggest an alternative explanation, whereby information near to a category boundary is processed with enhanced gain in the dmPFC and insular cortex.

## Materials and Methods

### Participants

Twenty right-handed volunteers (reporting normal or corrected-normal vision and no history of neurological problems), aged between 20 and 35 (9 females, 11 males), provided informed consent and were paid £30 compensation for taking part. The study was approved by local ethics committees.

### Stimuli

Stimuli were created and displayed using PsychToolBox (www.psychtoolbox.org) for MATLAB (Mathworks). Stimuli were presented on a custom shielded Samsung 40″ LCD screen (LTA400HF1) at a distance of 240 cm. On each trial participants viewed an array of 8 elements (colored shapes) circularly arranged (radius ∼3° visual arc) around a white central fixation point (5 pixels radius). Elements were equally spaced, equiluminant, and covered an equal area on the screen (width = height = 50 pixels for pure circle). Stimuli were presented on an equiluminant gray background. Each element was defined by a shape parameter (*S*) that determined its position on a continuous transition between a square (*S* = −1) and a circle (*S* = +1), and a color parameter (*C*) that determined its position on a continuous transition between blue (*C* = −1) and red (*C* = +1). This is described in detail elsewhere (de Gardelle and Summerfield 2011). On each trial, the parameter values for each dimension were drawn independently from a Gaussian distribution with mean *μ* and standard deviation *σ*. To ensure equal precision of the mean in all conditions, resampling occurred until the sampled trial *μ* and *σ* fell within a tolerance of 0.1% of the desired values.

### Design

The mean *μ* could take 1 of 4 values: 2 either side of the category boundary, giving rise to 2 absolute distances to category boundary, |*μ*|, which we refer to as low-mean versus high-mean conditions. The standard deviation *σ* was manipulated in 3 levels. The shape and color dimension were manipulated independently within and across trials. The task involved only 1D (either color or shape), varied across different blocks, such that each dimension could in turn be relevant or irrelevant for the decision. This afforded us a 2 (relevant |*μ*|) × 3 (relevant *σ*) × 2 (irrelevant |*μ*|) × 3 (irrelevant *σ*) within-participant factorial design.

### Thresholding

To equalize difficulty across participants and shape and color tasks, we used an adaptive procedure, in which the mean parameter of the array was varied to achieve an accuracy of 75% (low-mean condition) or 85% (high-mean condition). The 3 levels of variance were identical for all participants (0.1, 0.2, and 0.3). Each participant completed this staircase procedure (4 blocks: low and high mean condition in both tasks, with 144 trials in each block) on a standard testing PC on a day prior to the scanning session.

### Task and Procedure

**Figure 1.**
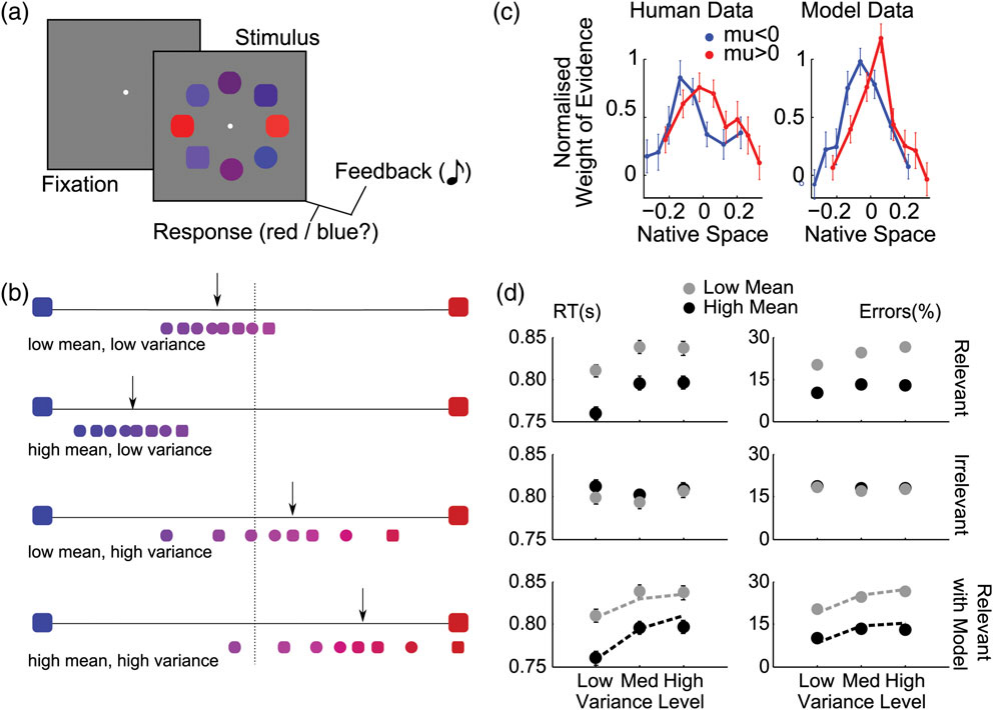
Task, behavior and modeling. (*a*) Schematic representation of a trial: a white central fixation point, was followed after 500 ms by the stimulus array, which participants categorized based on either the average shape (square vs. circle) or average color (red vs. blue) across all elements, with auditory feedback. (*b*) Example distributions of array elements for different combinations of mean and variance trials as labeled. The *x*-axis shows the feature space for the color task, with the larger blue and red squircles at each extreme representing color values of −1 (blue) and +1 (red). The smaller squircles show example stimuli for the color task drawn from a distribution with low mean, low variance (upper panel), high mean, low variance (upper middle panel), low mean, high variance (lower middle panel), and high mean, high variance (lower panel). The black arrow indicates approximate location of the distribution mean and the central dashed line is the category boundary. Thus, in these example trials arrows to the left indicate of the central line indicate that the correct response is “blue” and arrows to the right of the boundary should elicit a “red” response. (*c*) Coefficients from a logistic regression in which decision-relevant values, “ranked” in each trial, predicted observers’ choices, separately for blue/square (blue) and red/circle (red) stimulus arrays. Higher decision weights appear for inlying versus outlying elements. The abscissa indicates the average decision value of the elements in each rank, in native space. (*d*) Response times (RTs, left panels) and error rates (right panels), as a function of the mean and variance manipulations. Low mean and high variance correspond to high uncertainty. Top row: effect of the task-relevant manipulations. Middle row: effect of the irrelevant dimension. Bottom row: best-fitting model data (dashed lines) overlaid on the human data for the task-relevant manipulations. The model was fitted to errors only, and RTs are predictions.

On each trial, observers classified a circular array of 8 elements (squircles) according to their average color or shape (Fig. 1*a*). As described above, each of the 8 elements took on a color value (red to blue) and shape value (square to circle), both parameterized in the range −1 to 1, with the category boundary falling at zero (Fig. 1*b*). Color and shape were deemed decision-relevant in alternating blocks and the observers' task was thus to respond circle/square or red/blue according to whether the average feature value on the “relevant” dimension was greater or less than zero (ignoring the “irrelevant” dimension).

As shown in Figure 1*a*, the stimulus array appeared 500 ms after the onset of central fixation point, and remained on the screen for 1500 ms, during which time participants judged the average shape or color of the elements in the array, depending on the task block. Responses were made by pressing 1 of 2 keys on a button box (scanner) or computer mouse (thresholding task). In the scanner, participants used the index fingers of both hands to make responses whilst for thresholding the index and middle fingers of the same hand were used. Response mappings were fully counterbalanced across both dimensions between participants. At array offset, auditory feedback indicated response accuracy on each trial. Two ascending tones (400–800 Hz, 100 ms each) indicated a correct response while 2 descending tones (800–400 Hz, 100 ms each) were given for incorrect responses or misses (no-response trials). In the scanner, a jitter of 4 ± 2 s was introduced into the interstimulus interval (ISI); for the thresholding task, the ISI was jittered uniformly around 1 s (min: 0.85 s, max: 1.15 s).

Each block began with an instruction screen indicating the relevant decision dimension (either shape or color) and the response mapping for the block. Response mappings were fully counterbalanced across participants. Participants underwent 2 blocks of 144 trials for each task, for a total of 576 trials, and the order of tasks was pseudorandomized.

### Eyetracker: Acquisition

An MRI compatible eyetracker (Eyelink 1000 tracking device; SR Research, Ontario, CA, USA) was used to monitor eye movements in the fMRI scanner. The tracker was adjusted before each experimental block with a calibration/validation procedure in which participants followed with their eyes a small circle moving between 9 locations on the screen. Due to the difficulties of eyetracking in a scanner environment, accurate calibrations were not always possible and so eye data are presented from 10 participants. Data were collected using the PsychToolBox Eyelink toolbox and analyzed using in-house customized Matlab scripts. After downsampling to 200 Hz, we calculated the mean and variance of the displacement of the eye on each sample. Analyses of variance (ANOVAs) were then used to compare these estimates for different levels of stimulus array mean and variance.

### Behavioral Analyses

For each participant, we calculated accuracy (percent correct) and response latencies on the correct choices, in the 3 relevant variance × 2 relevant mean conditions of our design, and carried ANOVAs at the group level (Fig. 1*d*). The same procedure was used to analyze the effects of the irrelevant dimension.

We also calculated a weighting profile across elements (Fig. 1*c*), as in our previous work (de Gardelle and Summerfield 2011). This weighting profile is a plot of regression weights describing the contribution of each element to the trial decision. For each participant, we fit a probit regression in which the weighted sum of the 8 relevant feature values plus a constant term predicted the probability of “positive” choices, on a trial-by-trial basis. We sorted the 8 values in each trial before including them as predictors, so that the weights (i.e., the regression coefficients) corresponded to the different ranks along the task-relevant dimension. Then, we divided all weights in each participant by their root mean square, a normalization procedure that minimized the influence of unreliable estimates and that was neutral with respect to the weighting profile. We then compared the average normalized weights for the 4 outlying elements (i.e., the elements ranked 1, 2, 7, 8 in the trial) and the 4 inlying elements (ranked 3, 4, 5, 6), in a paired *t*-test across participants.

### Computational Model

As in our previous experiments (de Gardelle and Summerfield 2011), we calculated for each participant and task the proportion of trials in which a particular color or shape value *x* was associated via feedback with the left-hand response category (*P*(*L*|*x*)) or the right-hand response category (*P*(*R*|*x*)). Taking the logarithm of the ratio between these probabilities, we transformed the stimulus value *x* into a LPR value (log probability ratio; eq 1). This LPR value quantifies the association between *x* and the 2 response categories.

(1) $$\text{LPR}(x)=log\left(\frac{P(R|x)}{P(L|x)}\right)$$

Here, we calculated these probabilities in 10 bins along the *x*-axis (each bin contained 10% of the data), and fit them with a sigmoidal function. From then, we simulated a diffusion model in which the mean LPR over the 8 elements was used to drive the accumulation of evidence towards choice (de Gardelle and Summerfield 2011). The diffusion model had 2 free parameters for each participant: the noise in the diffusion and the amount of accumulated evidence needed to trigger the response. These parameters were optimized for the model to fit the 6 error rates for each participant and task. The simulated RTs (calculated in cycles) were then linearly scaled to the range of human RTs (in seconds), by setting the cycle duration such that the human and simulated RTs had the same standard deviation across all trials, and by adding a constant offset such that the simulated and human RTs had the same mean across all trials. This rescaling ensured that we could compare simulated and human RTs, while being neutral with respect to the profile of RTs across conditions.

### fMRI Acquisition and Preprocessing

Images were acquired in a 3-Tesla Siemens TRIO with a 32-channel head coil using a standard echo-planar imaging (EPI) sequence. Images were 64 × 64 × 36 volumes with voxel size 3 × 3 × 3 mm; acquired with a 2-s repetition time (TR) and 30-ms echo time. Four runs of 412 volumes were obtained, each of which lasted approximately 15 min and corresponded to 1 experimental block of 144 trials.

Preprocessing of the imaging data included correction for head motion and slice acquisition timing, followed by spatial normalization to the standard template brain of the Montreal Neurological Institute (MNI brain). Images were resampled to 3-mm cubic voxels and spatially smoothed with a 10-mm full width at half-maximum isotropic Gaussian kernel. A 256-s temporal high-pass filter was applied in order to exclude low-frequency artifacts. Temporal correlations were estimated using restricted maximum likelihood estimates of variance components using a first-order autoregressive model. The resulting nonsphericity was used to form maximum likelihood estimates of the activations.

### fMRI Analyses

All fMRI analyses were carried out using SPM8. SPM orthogonalizes regressors by default, but we ensured that this feature was turned off. We analyzed the data in 2 distinct ways. In “native space” analyses, we created independent regressors encoding the parameters |*μ*| and *σ* of the stimulus array. In “decision space” analyses, we substitute these for their counterpart in terms of the LPR-transformed feature values for each array, which we denote *U*_Mr_ and *U*_Vr_ (the subscript r indicates that this is about the task-relevant dimension). The decision space values represent the output from a proposed stage of processing in which the feature values (in color or shape space) are passed through a sigmoidal function. Including this transformation accounts for the behavioral finding of a weighting function in which outlying elements are downweighted compared with those at the center of the trial distribution of feature values.

(2) $$\text{mLPR}=\frac{1}{8}\times \sum_{k=1}^{8}\text{LPR}(x_{k})$$

(3) $$\text{vLPR}=\frac{1}{8}\times \sum_{k=1}^{8}(\text{LPR}(x_{k})-\text{mLPR}{)}^{2}$$

(4) $$U_{\mathrm{Mr}}=-|\text{mLPR}|$$

(5) $$U_{\mathrm{Vr}}=\sqrt{\text{vLPR}}$$

We calculated the decision space values as described in eq 2–5. First, we calculate the mean (mLPR) and variance (vLPR) of the LPR over the 8 elements (eqs. 2 and 3). Then, we define *U*_Mr_ and *U*_Vr_ (eqs. 4 and 5) such that both quantities positively scale with the intrinsic difficulty of the stimulus in the categorization task, which increases when the mean evidence approaches zero or when the feature values become more variable. These quantities were then used as predictors for the BOLD responses. In a separate analysis, we substituted the variability of the evidence for a different regressor encoding the sum across elements of the absolute value of the LPR.

Both native space and decision space analyses included the mean and variance regressors for the relevant and irrelevant dimensions (1–4), as well as separate regressors encoding (5) the feedback coded positive for correct and negative for incorrect trials, and nuisance regressors encoding movement parameters estimated from the realignment phase (7–12). These analyses were carried out independently for each participant, and the resulting *t*-statistics for each regressor were then subjected to *t*-tests at the group level. Voxels reported are those that survived at an uncorrected threshold of *P* < 0.0001. Full details of these voxels can be found in Tables 1 and 2.

**Table 1:** Voxels correlating with *U*_Mr_ in the decision space analysis at a threshold of *P* < 0.001 uncorrected for clusters larger than 20 voxels

Cluster *P* (FDR-corrected)	Cluster equivk	Peak *P* (FDR-corrected)	Peak *T*	Peak *x y z* (mm)
0.000	126	0.153	6.22	−34 −48 50
0.000	290	0.153	6.20	34 24 −2
		0.466	4.90	50 8 26
0.001	69	0.153	6.11	−30 28 −2
0.000	101	0.207	5.77	−46 4 2
0.002	57	0.382	5.22	10 24 42
0.002	56	0.560	4.64	34 −64 46
		0.561	4.40	38 −44 54
0.045	24	0.561	4.40	−50 32 30
		0.596	4.12	−46 44 22
		0.667	3.98	−38 28 18
0.017	33	0.078	6.70	66 −12 −14
0.000	120	0.294	5.13	−2 44 −2
0.000	101	0.294	5.12	−2 −52 30

Note: STATISTICS: *P*-values adjusted for search volume.

The following abbreviations have been used for the headings above and for all subsequent tables: Cluster *P*(FDR-cor): clusterwise *P*-value with false discovery rate correction for multiple comparisons; Cluster equivk: number of voxels in cluster; voxel *P* (FDR): voxelwise *P*-value with false discovery rate correction for multiple comparisons; peak *T*; voxelwise *t*-value; *x*, *y*, *z* ({mm)}: coordinates for the peak voxel, from the template brain of the Montreal Neurological Institute

**Table 2:** Voxels correlating with *U*_Vr_ in the decision space analysis at a threshold of *P* < 0.05 corrected (FDR) for clusters larger than 20 voxels

Cluster *P* (FDR-corrected)	Cluster equivk	Peak *P* (FDR-corrected)	Peak *T*	*x y z* (mm)
0.005	57	0.349	5.73	6 32 42
0.023	36	0.349	5.73	50 −28 −2
0.000	119	0.007	8.15	−30 −84 14
		0.013	7.04	−34 −84 6
0.000	331	0.011	7.37	34 −76 14
		0.053	5.90	26 −56 54
		0.053	5.86	22 −52 46
0.000	107	0.069	5.56	−18 −60 54

Note: Abbreviations as previously. STATISTICS: *P*-values adjusted for search volume.

In order to plot activity at different regions for the relevant and irrelevant mean and variance, we placed a sphere of 5 mm radius on the peak voxel in each cluster identified as responding to either the relevant mean or relevant variance. We used a spherical ROI centered on the peak voxel, rather than functionally-defined ROI, to avoid having to select a statistical threshold above which to include voxels. We then plotted its response to relevant and irrelevant mean and variance. We report statistics for (only) the response within this region to the orthogonal factor, that is, to the mean when data were extracted on the basis of the whole-brain search for voxels responsive to variance, and the variance when data were extracted on the basis of the whole-brain search for voxels responsive to mean. Because the regressors for the mean and variance (either |*μ*| and *σ* or the mean and standard deviation of LPR values) were orthogonal by design, this approach avoids any circularity or “double-dipping”.

In order to show hemodynamic response functions (Fig. 3*d*), a further whole-brain finite impulse response (FIR) analysis was run with the native-space values. This model replicated the structure of the main analyses, including the mean and variance of both the relevant and irrelevant dimension. In this analysis, 16 time bins (each corresponding to 2 s) were entered into the design matrix for each regressor described above. Positive and negative feedback, as well as movement parameters, were also included in the design matrix for this analysis. Data in Figure 3*d* are presented from a sphere of 5 mm radius centered on the peak voxel within the dmPFC ROI.

## Results

### Behavioral Data

Pre-experimental calibration ensured comparable performance in shape and color tasks for the fMRI experiment (see Materials and methods). Mean error rates (shape: 18%, color: 16%) and response times (shape: 800 ms, color: 803 ms) were not different across tasks. ANOVAs revealed main effects of both mean (i.e., proximity to category boundary) [*F*_1,20_ = 92.7, *P* < 0.001] and variance (i.e., element heterogeneity) [*F*_2,40_ = 18.0, *P* < 0.001] on correct response latencies (Fig. 1*d*, top left panel), but no significant interaction [*F*_2,40_ = 0.46, *P* < 0.58]. Comparable effects were found for accuracy (Fig. 1*d*, bottom left panel), with more errors occurring on trials with low mean than high mean [*F*_1,20_ = 104, *P* < 0.001], or trials with high variance versus low variance [*F*_2,40_ = 7.80, *P* < 0.001], and no interaction [*F*_2,40_ = 2.70, *P* < 0.15]. The irrelevant mean and variance had no significant effect on RT or errors (all *P*-values > 0.05; Fig. 1*d*, right panels). Analyses of eye movements (mean length and variance of the eye path) confirmed that they did not differ between conditions (all *P* > 0.19). Together, these data confirm the previous finding that array mean and variance have independent (i.e., noninteracting) effects on behavior (de Gardelle and Summerfield 2011).

When decisions involve integration of multiple independent sources of evidence, the question additionally arises of how much each source affects the final choice. In a previous report (de Gardelle and Summerfield 2011), we demonstrated that elements that were outlying in value space (e.g., in a red/blue categorization, the 2 bluest or reddest elements amongst the 8 items in a trial) carried less weight in the choice than inlying elements (e.g., the 4 central elements on the red/blue axis), a phenomenon termed “robust averaging.” Calculating the weight that each element (sorted by its rank) carried in the choice using logistic regression (see Materials and methods), we replicated this finding here. We observed significantly higher weights (regression coefficients) for inlying versus outlying items [*t*_20_ = 2.72, *P* < 0.013], when collapsing across relevant feature dimension (color or shape). These data are shown independently for relevant feature dimension in Figure 1*c* (left panel).

### Computational Model

We have previously shown that it is possible to account for both this “robust averaging” phenomenon and prolonged RTs for more variable arrays if decision values are “squashed,” for example, by being passed through a soft-threshold nonlinearity (i.e., a sigmoid function) before being averaged and integrated across time (de Gardelle and Summerfield 2011). One computationally parsimonious manner of implementing this transformation is to recode the feature value of each element according to the logarithm of the probability ratio (LPR) between the 2 response options, given the history of feedback for this stimulus value. This transformation mirrors what might occur in a simple, biologically plausible neural network in which inputs are mapped onto binary responses via weights updated with a supervised, winner-takes-all rule (Ratcliff et al. 1999).

For each element *x*, we thus defined the LPR value of *x* (see eq 1 and Materials and methods) which expresses the probabilistic evidence conveyed by the stimulus *x* in favor of the rightward option (for positive LPR values) or the leftward option (for negative values). In the context of our experiment, this LPR transformation had a sigmoidal shape, by which extreme elements do not “pull their weight” compared with those near the center of feature space (where the function is roughly linear). Of note, the likelihood function is sigmoidal in our experiment because of the mixture of Gaussians (4 levels of mean × 3 levels of variance) from which samples were drawn across trials. Consequently, using the average LPR to drive a drift-diffusion decision process could account for the slowing down of response latencies on more variable arrays, because the weight of outlying evidence is muted, reducing the overall input to the decision process on those trials (Fig. 1*c*, lines). In addition, this LPR transformation could capture the downweighting of elements with extreme feature values exhibited in participants' behavior (Fig. 1*c*, right panel). These findings replicate those of our earlier work (de Gardelle and Summerfield 2011).

In what follows, we assess how brain responses of participants can be predicted by the intrinsic uncertainty of each stimulus array in the categorization task. To do so, we defined the quantities *U*_Mr_ and *U*_Vr_ to express the effects of manipulating the stimulus mean and variance in terms of the “decision-space,” that is, the space of the LPR-transformed values (see eqs 3–6 and Materials and methods).

### BOLD Responses Associated with Mean Evidence

In the first set of analyses, we thus searched for voxels where the BOLD signal correlated positively with the quantity *U*_Mr_ which reflects the uncertainty due to the mean of the evidence on the task-relevant dimension. In what follows, we report uncorrected statistics but all reported activations exceeded a cluster-corrected threshold of *P* < 0.05 unless explicitly noted in the text.

**Figure 2.**
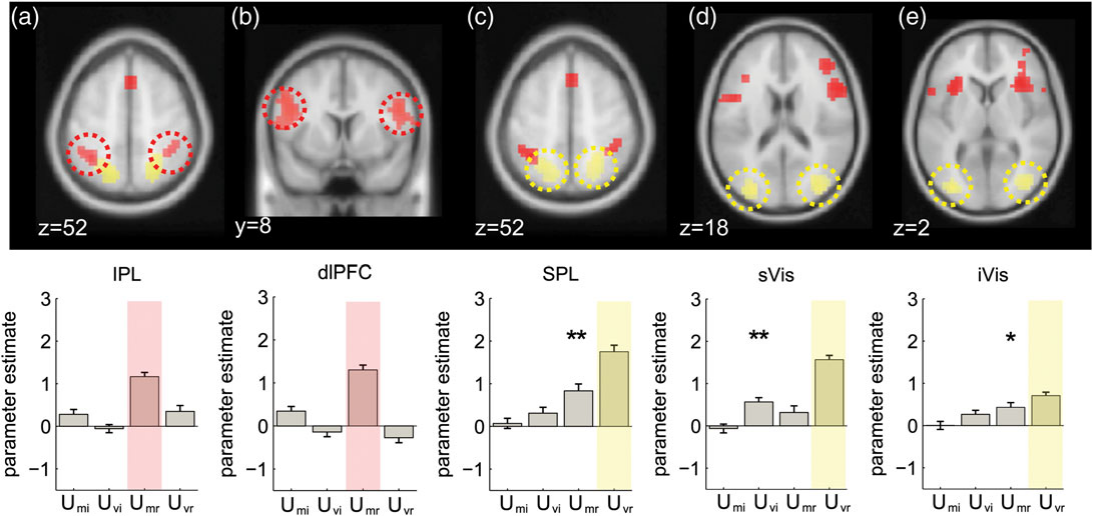
Imaging results from dorsolateral prefrontal, parietal and visual cortices. Top row: voxels where BOLD activity was responding to positively correlated with mean-related uncertainty (*U*_Mr_, in red), and positively correlated with variance-related uncertainty (*U*_Vr_, in yellow). All activations are rendered on the template brain of the Montreal Neurological Institute with an uncorrected threshold of *P* < 0.001 (see text and tables for full list of activation and peak coordinates). Bottom row: average parameter estimates from a 5 mm sphere centered on the peak activation from the cluster highlighted with a dashed ellipse, for regressors encoding uncertainty due to the mean and variance for relevant and irrelevant dimensions. Stars indicate significance: *P < 0.05, **P < 0.01, ***P < 0.001. Red and yellow shading denotes the condition used to define the ROI. (*a*) Voxels in the inferior parietal cortex (IPL) responding to *U*_Mr_. (*b*) Voxels in the dorsolateral prefrontal cortex (dlPFC) responding to *U*_Mr_ (*c*) superior parietal cortex (SPL) showed a positive correlation with *U*_vr_ (and *U*_Mr_, see bar plot). (*d* and *e*) We subdivided a large region of visual cortex showing activity positively correlated with *U*_vr_ into superior (sVis) and inferior (iVis) regions.

**Figure 3.**
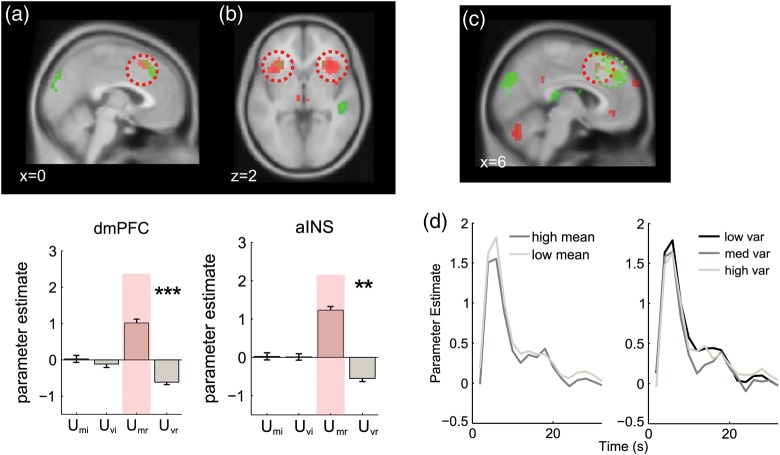
Imaging results from dorsomedial prefrontal cortex and anterior insula. (*a*) Upper panel: voxels responding positively to uncertainty due to the mean (*U*_Mr_; red) and negatively to uncertainty due to the variance (*U*_Mr_; green) rendered onto a sagittal slice of the MNI template brain. The corresponding bar plot shows mean responses extracted a sphere of 5 mm radius around the peak voxel for the highlighted cluster, with stars denoting the statistical significance as in Figure 2. (*b*) Same results for an axial slice showing the AINS. (*c*) Correlations with native space mean (positive correlation with |μ|, red) and standard deviation (negative correlation with *σ*, green) in dmPFC, rendered onto a sagittal slice at a threshold of *P* < 0.005 uncorrected. The scale indicates the *t*-value. (*d*) Left panel: hemodynamic response functions (HRFs) generated from a finite impulse response (FIR) model for the dmPFC ROI (5 mm sphere extracted from peak of native space activation) for low mean (i.e., |*μ*| close to category boundary; light gray) and high mean (dark gray). *X*-axis shows time in scans (2 s). Right panel: HRFs for low (black), medium (dark gray), and high (light gray) variance.

The results are shown in Figures 2 and 3 (red blobs) and reported in Table 1. *U*_Mr_ was positively associated with the BOLD signal in the dorsomedial prefrontal cortex (dmPFC; Fig. 3*a*), [peak: 0, 22, 48; *t*_(20)_ = 5.22, *P* < 0.0001] and anterior insular cortex (AINS; Fig. 3*b*) [left peak −30, 20, 2; *t*_(20)_ = 6.07, *P* < 0.00001; right peak: 34, 24, −2; *t*_(20)_ = 6.2, *P* < 0.00001;]. Note once again that this positive correlation signals higher BOLD signals as LPR approaches zero and responses come into conflict. A positive association with *U*_Mr_ was also observed in the inferior parietal lobule (IPL) [left peak: −34, −48, 50; *t*_(20)_ = 6.22, *P* < 0.00001; right peak: 38, −44, 54; *t*_(20)_ = 4.40 *P* < 0.001; Figure 2*a*, far left] and dorsolateral prefrontal cortex (dlPFC) both anteriorly in Brodmann's area 46 [left peak: −50, 32, 30, *t*_(20)_ = 4.40, *P* < 0.0002; right peak: 42, 44, 26, *t*_(20)_ = 4.76, *P* < 0.0001) and more caudally in Brodmann's area 8 [left peak: −46, 4, 26 *t*_(20)_ = 5.77, *P* < 0.0001; right peak: 50, 8, 26; *t*_(20)_ = 4.90, *P* < 0.0001; Figure 2*a*, center left]. Negative correlations with *U*_Mr_ (i.e., higher BOLD with growing unsigned LPR) were observed in the ventromedial prefrontal cortex and posterior cingulate (not shown).

### BOLD Responses Associated with Evidence Variability

Our next step was thus to assess whole-brain responses to the variability of the evidence. As for the results reported above, all results shown remained significant following correction for multiple comparisons at the cluster level (see Table 2). Here, we report brain regions that were sensitive, across trials, to the standard deviation of the LPR over elements; that is, *U*_Vr_ (uncertainty due to variability) as defined above.

Positive correlations with evidence variability (*U*_Vr_) were observed in the visual cortex (Fig. 2*d*,*e*, yellow blobs). These reached maxima in separate sites in the middle occipital gyrus (sVis; visual area 3) which were positively activated when the evidence was more variable [left peak: −30, −84, 14 *t*_(20)_ = 8.15 *P* < 0.000001; right peak: 34, −76, 14, *t*_(20)_ = 7.37, *P* < 0.00001; center right panel] and at a more ventral site overlapping with the inferior occipital gyrus (iVis) [*t*_(20)_ = 4.02, *P* < 0.0004; far right panel]. We also observed strong positive correlations with evidence variability at sites coextensive with the superior parietal lobule (SPL) [left peak: −18, −60, 54, *t*_(20)_ = 5.56, *P* < 0.0001; right peak: 26, −56, 54, *t*_(20)_ = 5.90, *P* < 0.00001; middle panel].

As shown in Figure 3*a* (green blobs), however, negative correlations with *U*_Vr_ were observed in the dorsomedial prefrontal cortex (6, 32, 42, *t*_(20)_ = 5.73, *P* < 0.00001;). We also observed negative correlations with evidence variability in the anterior insula, with symmetric peaks in the left (−30, 24, −2, *t*_(20)_ = 3.95, *P* < 0.001) and right (26, 20, 2, *t*_(20)_ = 3.95, *P* < 0.001) hemispheres, although these fell just short of corrected statistical thresholds (Fig. 3*a*, right). These negative correlations with *U*_Vr_ denote voxels where the BOLD response increased as arrays became more homogenous.

### BOLD Response to the Mean and Variance of the Irrelevant Dimension

Our stimulus array consisted of a relevant and an irrelevant dimension (either shape or color). This afforded us the opportunity to carry out precisely parallel analyses on the dimension of the array which was irrelevant to the decision. One small cluster was found to negatively correlate with *U*_Mi_ (*t*_(20)_ = 4.23, *P* < 0.001), but it did not survive correction for multiple comparisons. No activations were observed in any regions correlating with the variance of the “evidence” on the irrelevant dimension (*U*_Vi_), although positive correlations with the irrelevant variance were observed in the visual cortex at very lenient thresholds (*P* < 0.005 uncorrected). The lack of reliable correlation with the statistics of the irrelevant dimension confirms that the above-described effects are due to processing of decision-relevant signals.

### Overlapping and Nonoverlapping Responses to Evidence Mean and Variability

To compare responses to mean and variance, we extracted regions of interest focused on peaks responding positively to *U*_Mr_ and tested their sensitivity to evidence variability (i.e., *U*_Vr_), and vice versa (Fig. 2*b*). ROIs were defined as a 5 mm radius sphere centered on the peak activated voxel in each cluster. Of note, these analyses were strictly independent from one another, as the mean and standard deviation of the feature values in the array were orthogonal by design, and remained so after conversion to LPR values (mean *r* = −0.01, *P* = 0.29, *t*-test of Fisher's *z*-scores against zero). For completeness, we additionally verified sensitivity to the irrelevant mean and variance via this approach, making separate plots for *U*_Vr_ and *U*_Mr_ (correlations with the task-relevant variables) and *U*_Vi_ and *U*_Mi_ (correlations with the task-irrelevant variables; see also Fig. 2*b*).

Voxels responsive to *U*_Mr_ in the IPL and dlPFC failed to respond to evidence variability (all *P*-values > 0.05; Fig. 2*b* far and center left panels). However, superior parietal lobule regions sensitive to *U*_Vr_ were additionally responsive to the *U*_Mr_, that is, to the proximity of the mean LPR to zero, in both hemispheres [left peak, *t*_(20)_ = 2.334, *P* = 0.02; right peak, *t*_(20)_ = 2.732, *P* = 0.007] (Fig. 2*c*). Alone among these ROIs, the more superior visual region responded additionally to the task-irrelevant variance *U*_Vi_ (Fig. 2*d*), whereas the more ventral visual region responding to variance also responded to *U*_Mr_ [*t*_(20)_ = 1.80, *P* < 0.04] (Fig. 2*e*).

By contrast however, peak voxels in the dorsomedial PFC and anterior insula identified by virtue of their response to mean evidence were additionally responsive to evidence variance (Fig. 3). Negative correlations with *U*_Vr_ were observed in the dmPFC (*t*_(20)_ = 5.16, *P* < 0.00003; Fig. 3*a*) but also reliable in the right (*t*_(20)_ = 2.44, *P* < 0.012) and left (*t*_(20)_ = 2.88, *P* < 0.005) AINS (Fig. 3*b*). Note once again that negative correlations with *U*_Vr_ signal increasing BOLD signal as the array becomes more homogenous.

These results are striking for 2 reasons. First, they show a sharp dissociation between 2 portions of a network frequently activated by decision uncertainty or the demand of action selection. Parietal sites responded to the level of relative evidence in the stimulus array—quantified as the proximity of the LPR to zero—but either failed to respond (at inferior sites) or responded positively (at superior sites) to the variability of the evidence. Medial prefrontal and insular sites, by contrast, were equally responsive to the LPR, but responded negatively to the variability of the evidence. In other words, more positive-going BOLD signals were observed in the dmPFC/AINS when the stimulus array was more homogenous. This is particularly surprising, because the dmPFC in particular has been proposed to be sensitive to the likelihood of an error (Brown and Braver 2005) or to time-on-task (Grinband et al. 2011), whereas here participants were both faster and more accurate on trials with more homogenous feature values. We thus sought to validate these findings with a further series of control analyses.

### Correlations with Mean and Variance of Raw Feature Values

Could the negative correlation between dmPFC and AINS BOLD signal and evidence variability be due to some artifact of our log-probability transform of decision values? To rule out this possibility, we conducted the same analyses as described above but using the statistics of raw (native space) feature values |*μ*| and *σ* rather than their LPR-transformed counterparts. Globally, the results were qualitatively similar, but statistically more modest. In Figure 3*c*, we show the overlapping clusters responding positively to mean-related uncertainty (negative correlation with |*μ*|) and negatively to variance-related uncertainty (positive correlation with *σ*) in the medial PFC for decision-space and native-space analyses. Similar results were obtained for the AINS and visual and parietal cortices.

### Hemodynamic Response Functions

To additionally ensure that our unexpected findings were not due to misfitting of our basis function (canonical hemodynamic response) to the data, we reanalyzed our whole-brain data using a FIR filter (which makes no assumptions about the shape of the BOLD response) and plotted the HRFs for low and high mean |*μ*| (Fig. 3*d*, left), and low, medium, and high standard deviation *σ* (Fig. 3*d*, right) separately (i.e., in native feature space). The peak BOLD response averaged across 4 and 6 s poststimulus onset confirmed the pattern of previous analyses, with larger responses to smaller values of |*μ*| (*t*_(20)_ = 2.414, *P* < 0.0127) and larger responses on trials with low values of *σ* (*t*_(20)_ = 2.390, *P* < 0.014). There was no interaction between mean and variance observed.

### Voxelwise Correlations Between Effects of Mean and Variance

**Figure 4.**
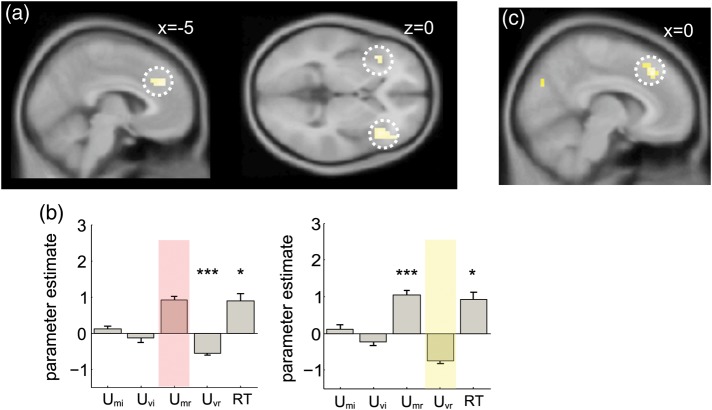
Additional imaging results (*a*) voxels where there was a significant negative correlation between the response to *U*_Mr_ and the response to *U*_Vr_ shown on a sagittal (left panel) and axial (right panel) slice at a threshold of *P* < 0.001 uncorrected. (*b*) Results of an analysis in which reaction time (RT) was included in the design matrix. Bar graphs show parameter estimates for response to the mean and variance of task-irrelevant (*U*_Mi_ and *U*_Vi_) and task-relevant values (*U*_Mi_ and *U*_Vi_) and reaction time (RT), for a dmPFC region of interest defined by its significant response to *U*_Mr_ (left panel) and *U*_Mv_ (right panel). Stars indicate significance: *P < 0.05, **P < 0.01, ***P < 0.001. Note positive correlation with RT in each region, and that all effects described persist even once RT is included. (*c*) Voxels showing a negative correlation with *D*, indexing the absolute distance to bound of all of the elements in the array, rendered on a sagittal slice at a threshold of *P* < 0.001 uncorrected.

Our statistical approach involved identifying voxels that responded to evidence mean and testing their sensitivity to evidence variability, and vice versa. One limitation of this approach is that 2 adjacent but nonoverlapping clusters might become smeared into one by spatial smoothing, potentially giving rise to the spurious impression that a single region responds to both variables. We thus conducted a further analysis in which we correlated the response to *U*_Mr_ and *U*_Vr_ in a voxelwise fashion using the unsmoothed data, and converted the correlation coefficients at each voxel to a Fisher's *z*-score, permitting parametric statistics at the group level. The resulting group statistical maps, which were only smoothed after correlations were calculated, indicated voxels where there were significant correlations between the response to *U*_Mr_ and *U*_Vr_. The results are shown in Figure 4*a*,*b*. We observed a cluster of negative correlation between response to these 2 variables in the vicinity of the dmPFC/ACC, with peaks at −2, 28, 30 (*z* = 3.17, *P* < 0.001, uncorrected) and −6, 36, 30 (*z* = −3.34, *P* < 0.0005, uncorrected; Fig. 4*a*, left). Additional clusters were observed at the left (−38, 20, −6, *z* = −3.43, *P* < 0.0003, uncorrected) and right (42, 24, −2, *z* = −3.32, *P* < 0.0005, uncorrected) AINS (Fig. 4*a*, right). Within the ROI defining, the dmPFC by its sensitivity to *U*_Mr_, the average Fisher's *z*-score was also significant (*z* = −2.39, *P* < 0.009). In other words, those voxels that responded positively to *U*_Mr_ (higher signal as the mean LPR approached zero) tended to respond negatively to *U*_Vr_ (higher signal as evidence became more homogenous) and vice versa. No positive or negative correlations between response to *U*_Mr_ and response to *U*_Vr_ were observed in other regions sensitive to *U*_Mr_, such as the parietal cortex.

### Neural Correlation with Reaction Time in dmPFC

One reason why the negative correlation with *U*_Vr_ in the dmPFC is surprising is that this region has often been reported to exhibit a positive correlation with reaction time (RT), and here RTs are longer when feature variability is high. We thus conducted a further analysis in which a regressor whose height was parametrically modulated by RT on each trial was included. As expected, the dmPFC region (defined as above) correlated positively with RT (*t*_(20)_ = 2.38, *P* < 0.0139) as well as *U*_Mr_. Critically however, the inclusion of RT left the negative-going response to variability in the dmPFC region defined by positive correlation with *U*_mr_ intact (Fig. 4*b* and *t*_(20)_ = 5.27, *P* < 0.00002). In other words, the negative correlation between BOLD activity in the dmPFC and evidence variability persists even though the latter elicits greater behavioral cost.

### Correlation with Distance-to-Bound in the dmPFC

One explanation for this counterintuitive finding is that information that falls close to the category boundary is processed with enhanced gain. This proposal follows naturally from our modeling approach in which individual stimulus values were sigmoidally transformed and averaged across elements within the array, before they contribute to an “integration-to-bound” decision process (in this decision process, some information corrupted with noise is accumulated over time until a criterion is reached that triggers the response, see, e.g., Ratcliff and McKoon, 2008). One consequence of the sigmoidal transformation is that information closest to the boundary will have the most powerful impact on choices, as demonstrated by the “robust averaging” behavior observed in this cohort (and previously) whereby elements falling far from the boundary are downweighted in the choice.

One plausible neural implementation of this model is one in which the gain of encoding of decision information is strongest near to the category boundary. We thus attempted to formalize this idea, calculating a new quantity that indexed total distance of all elements to the category boundary, in decision space. We call this variable distance to bound (*D*).

$$D=\sum_{k=1}^{8}|\text{LPR}(x_{k})|$$

*D* thus indexes the total absolute divergence of the evidence from the category boundary, summed across the whole array. Under the conditions created by our experiment, *D* is very highly correlated with evidence variability (average *r* = 0.87) and uncorrelated with *U*_Mr_ (average *r* = 0.38 across the cohort).

For completeness, we conducted a separate analysis in which we used *D* and *U*_Mr_ to predict brain activity on a trial-by-trial basis (Fig. 4*c*). Because of the strong correlation between *D* and evidence variability, it is not surprising that we observed a robust negative response to *D* in the dmPFC (peak: 10, 28, 42, *t*_(20)_ = 4.94, *P* < 0.00004) in a whole-brain analysis. We additionally observed a significant negative response to *D* in the peak dmPFC voxel responsive to *U*_Mr_ (*t*_(20)_ = 3.71, *P* < 0.001). In other words, the dmPFC correlates negatively with the absolute distance of all elements to the decision bound, or conversely, correlates positively with proximity-to-bound.

## Discussion

Most laboratory-based categorization tasks require observers to classify a single, isolated element into 1 of 2 categories. Where evidence is ambiguous, observers equivocate; formal models capture these prolonged decision latencies with mutual inhibition between competing response nodes (Botvinick et al. 2001; Bogacz et al. 2006), or with overt mechanisms that put the brakes on responding in order to optimize performance (Aron et al. 2007; Forstmann et al. 2010; Cavanagh et al. 2011; Ratcliff and Frank 2012). Neuroimaging studies have attributed this function to a characteristic network of interconnected brain regions (including the dorsomedial prefrontal and anterior insular cortices) which are known to become active when competing options exhibit similar response values, and that predict the slow-down (Grinband et al. 2011) and increased error probability (Brown and Braver 2005) characteristic of these trials. However, these studies have tended to manipulate the degree of conflict between options, but have not formally dissociated the twin influences of mean-related and variance-related uncertainty (Huettel et al. 2005; Grinband et al. 2006; Bach et al. 2011). Other researchers have controlled the sensitivity of sensory discrimination judgments by adjusting the signal-to-noise ratio in a stimulus (e.g., the ratio of coherently to randomly moving dots in a random dot kinetogram), which varies uncertainty but precludes an assessment of the independent influences of evidence mean and evidence variability on choice (Ho et al. 2009; Liu and Pleskac 2011; Filimon et al. 2013).

Here, manipulating the mean and variability of evidence independently, we report a new finding that is hard to reconcile with current models emphasizing the role of the dmPFC in processing conflict (Botvinick et al. 1999), error likelihood (Brown and Braver 2005), or negative surprise (Alexander and Brown 2011), and that contradicts the view that dmPFC inevitably scales with time on task (Grinband et al. 2011). Irrespective of its mean value, when evidence is more heterogeneous (variable), reaction times, and errors increase, but the neural response in the dmPFC and AINS is the polar opposite: “less” activity for “more” variable evidence. This finding was observed both for raw (native-space) values of mean and standard deviation of the evidence, as well as following a log-probability scaling of these values, a transformation that allowed us to better account for the behavioral data. This finding places an important new constraint on models that have sought to link neural observations in these brain regions to computational-level descriptions of decisions made under uncertainty.

We begin by discussing the less controversial of our findings. Consistent with previous reports, BOLD signals in portions of the dlPFC and parietal cortex varied with uncertainty about which response to choose. In our study, this was formalized as the log probability ratio (LPR), a quantity that naturally expresses the relative evidence for one choice over another under an ideal observer framework, such as that underpinning signal detection theory (Green and Swets 1966) and the serial probability ratio test (Wald and Wolfowitz 1949)—in other words, the extent to which decision-relevant evidence prompts (and requires the resolution of) response conflict. Accordingly, a well-established previous literature links the demand provoked by choosing among competing responses with the activity of the lateral prefrontal cortex (Miller and Cohen 2001; Koechlin et al. 2003; Kerns et al. 2004; Egner and Hirsch 2005; Badre and D'Esposito 2007; Koechlin and Summerfield 2007). Similarly, firing rates of single neurons in the IPL scale with the evolving relative evidence in favor of one option over another (Roitman and Shadlen 2002; Yang and Shadlen 2007). An interpretation consistent with this extensive literature is that these signals reflect the demand of resolving conflict provoked by competing or conflicting evidence.

Behaviorally, performance suffers as array variance increases (de Gardelle and Summerfield 2011). A major aim of our study was to pinpoint the source of this cost in human information processing, and understand its neural basis. Standard decision theoretic models, such as those capturing the decision process as a particle diffusing towards a response boundary (Ratcliff and McKoon 2008), treat evidence as a scalar quantity, thereby sidestepping the question of how evidence is integrated from multiple sources, and offering only limited guidance for understanding the computational cost of evidence heterogeneity. Two theoretical accounts are nevertheless relevant for our study. The first is the conflict monitoring hypothesis proposed by Botvinick and colleagues (Botvinick et al. 2001; Yeung et al. 2004) according to which the dmPFC is sensitive to the cumulative product of the activation in all rival response units associated with a stimulus. Assuming that the activation evoked by a feature is proportional to its distance from the category boundary (e.g., during red/blue discrimination, that a clearly red stimulus elicits more activation in the relevant response unit than a purplish-red stimulus), then this theory incorrectly predicts greater dmPFC activity for more heterogeneous arrays, where there exists strong but contradictory evidence for the 2 opposing responses. A second theory is the view, attributable to Frank et al. (2007), that the dmPFC is instrumental in inhibiting prepotent actions when 2 conflicting responses are both favorable, for example, during “win/win” economic choices between 2 preferred goods. Presumably, this theory also incorrectly predicts heightened BOLD signals for more variable arrays, where the need to inhibit an impulsive response driven by a single outlying element would be greatest. Thus, to the best of our understanding, these theories cannot account for the current findings, and do not shed light on why the dmPFC does not encode the behavioral cost of high variance arrays.

The failure of our findings to accord with these well-motivated and well-supported accounts of dmPFC function surprised us, and prompts us to be cautious in discussing and interpreting our findings. Nevertheless, we wish to propose a tentative theory that does account for the current data, and simultaneously offers a new explanation of dmPFC function. We offer this theory because it may open potential new avenues for future research, but with the frank acknowledgement that it was formulated post hoc, to account for data that we did not predict a priori.

Our explanation begins with the empirical finding that judgments are more sensitive close to the category boundary, a well-described feature of human categorical perception (Shepard 1987; Tenenbaum and Griffiths 2001). This notion informs the computational simulations that account for behavioral data reported here and in our previous work: that prolonged RTs to more variable trials, and robust averaging, can be explained if feature information is transformed sigmoidally before being integrated to bound (de Gardelle and Summerfield 2011). The sigmoidal transfer function, whose steepest portion bisects the midpoint between the 2 categories, ensures that the information falling closest to the boundary is processed with the highest gain, and has the most impact on human choices. This sigmoidal shape explains perceptual magnet and perceptual categorization effects, by which perceptual similarity is increased within the same category and decreased for elements falling across the category boundary. Such a pattern can result from optimal inference schemes applied to categorization tasks (Bonnasse-Gahot and Nadal 2008; Feldman et al. 2009).

One possibility, thus, is that the increased BOLD signal observed at the dmPFC and AINS reflects the heightened gain associated with information falling closest to the boundary—such that dmPFC signals correlating strongly with the absolute distance-to-bound of the information in the visual array. For example, tuning functions might adapt over the course of the experiment, so that those closest to the boundary produce stronger output for a fixed input. An adapting transfer function for feature information is suggested by our previous work, in which outlier downweighting depended on the range of feature information available across the experiment (de Gardelle and Summerfield 2011). Other studies have suggested a specific increase in BOLD signal in sensory regions for stimuli falling close to a known categorical boundary, such as the cardinal axes for tilted gratings (Furmanski and Engel 2000). Here, we observed increases in BOLD signal observed in the dmPFC and AINS for information close to the category boundary, which might reflect a boost to an integration process occurring within these regions, or might be driven by inputs from interconnected regions, such as the parietal cortex.

Indeed, researchers have puzzled over the finding that increased decision information is associated with decreases in BOLD signal over regions that might play a key role in integration of evidence, such as the parietal cortex, AINS, and dmPFC, given that single-cell studies suggest net increases in firing as proximity to a choice, switch, or reward increases, at least in some neurons (Shidara and Richmond 2002; Gold and Shadlen 2007; Hayden et al. 2011). One idea that has been proposed is that when evidence is weak or ambiguous, accumulation is prolonged, and so the time-integral of firing will be greater under these circumstances (Basten et al. 2010). However, this theory predicts a reversal of the effect during an interrogation paradigm, when evidence is presented for a fixed period before a response is solicited—a finding that does not square with extant evidence (Huettel et al. 2005). The current proposal, in which increased BOLD signal on trials with weak or ambiguous evidence reflects the proximity of the decision information to the category boundary in those situations, offers an alternative explanation for this finding.

Outside of the frontal regions, yet another distinct pattern was observed in the visual cortex and more superior regions of the parietal cortex, where voxels responded positively to the variability of information in the array. Sensitivity to evidence variability in dorsal stream regions might serve a number of purposes. For example, the first 2 statistical moments of the perceptual evidence in a natural scene provide estimates of the range and central tendency of visual information across space, which might in turn allow the observer to adjust the gain of neuronal responding to deal with currently available information. Processing of evidence variability may also play a role in computing the gist of perceptual information, which can in turn guide saccadic exploration strategies (Torralba et al. 2006) and facilitate rapid decision-making, for example, by permitting divisive normalization (Carandini and Heeger 2012). In our experiment, the fact that evidence variability seemed to be associated with changes in neural activity relatively early in the processing stream (e.g., in visual regions) points to an early role in the choice process, albeit confined to task-relevant information. Investigations of evidence variability and the extraction of gist-like information in complex visual arrays may prove a fruitful area of future research.

## Funding

This work was supported by a European Research Council award to C.S. Funding to pay the Open Access publication charges for this article was provided by a Wellcome Trust Project Grant awarded to CS.
